# New Opportunities to Advance the Field of Sports Nutrition

**DOI:** 10.3389/fspor.2022.852230

**Published:** 2022-02-17

**Authors:** Kristin L. Jonvik, Michelle King, Ian Rollo, Trent Stellingwerff, Yannis Pitsiladis

**Affiliations:** ^1^Department of Physical Performance, Norwegian School of Sport Sciences, Oslo, Norway; ^2^Gatorade Sports Science Institute, PepsiCo Life Sciences, Barrington, IL, United States; ^3^Gatorade Sports Science Institute, PepsiCo Life Sciences, Global R&D, Leicestershire, United Kingdom; ^4^Canadian Sport Institute-Pacific, Victoria, BC, Canada; ^5^Exercise Science, Physical and Health Education, University of Victoria, Victoria, BC, Canada; ^6^School of Sport and Health Sciences, University of Brighton, Eastbourne, United Kingdom

**Keywords:** innovation, wearables, technology, performance, health, diet, wellness, athletes

## Abstract

Sports nutrition is a relatively new discipline; with ~100 published papers/year in the 1990s to ~3,500+ papers/year today. Historically, sports nutrition research was primarily initiated by university-based exercise physiologists who developed new methodologies that could be impacted by nutrition interventions (e.g., carbohydrate/fat oxidation by whole body calorimetry and muscle glycogen by muscle biopsies). Application of these methods in seminal studies helped develop current sports nutrition guidelines as compiled in several expert consensus statements. Despite this wealth of knowledge, a limitation of the current evidence is the lack of appropriate intervention studies (e.g., randomized controlled clinical trials) in elite athlete populations that are ecologically valid (e.g., in real-life training and competition settings). Over the last decade, there has been an explosion of sports science technologies, methodologies, and innovations. Some of these recent advances are field-based, thus, providing the opportunity to accelerate the application of ecologically valid personalized sports nutrition interventions. Conversely, the acceleration of novel technologies and commercial solutions, especially in the field of biotechnology and software/app development, has far outstripped the scientific communities' ability to validate the effectiveness and utility of the vast majority of these new commercial technologies. This mini-review will highlight historical and present innovations with particular focus on technological innovations in sports nutrition that are expected to advance the field into the future. Indeed, the development and sharing of more “big data,” integrating field-based measurements, resulting in more ecologically valid evidence for efficacy and personalized prescriptions, are all future key opportunities to further advance the field of sports nutrition.

## Introduction

**Figure 1:**
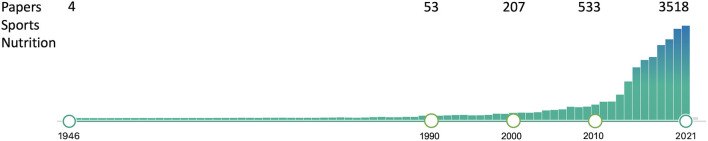
Publications in Pubmed using the search term “Sports Nutrition” as of December, 2021^1^.

**Figure 2:**
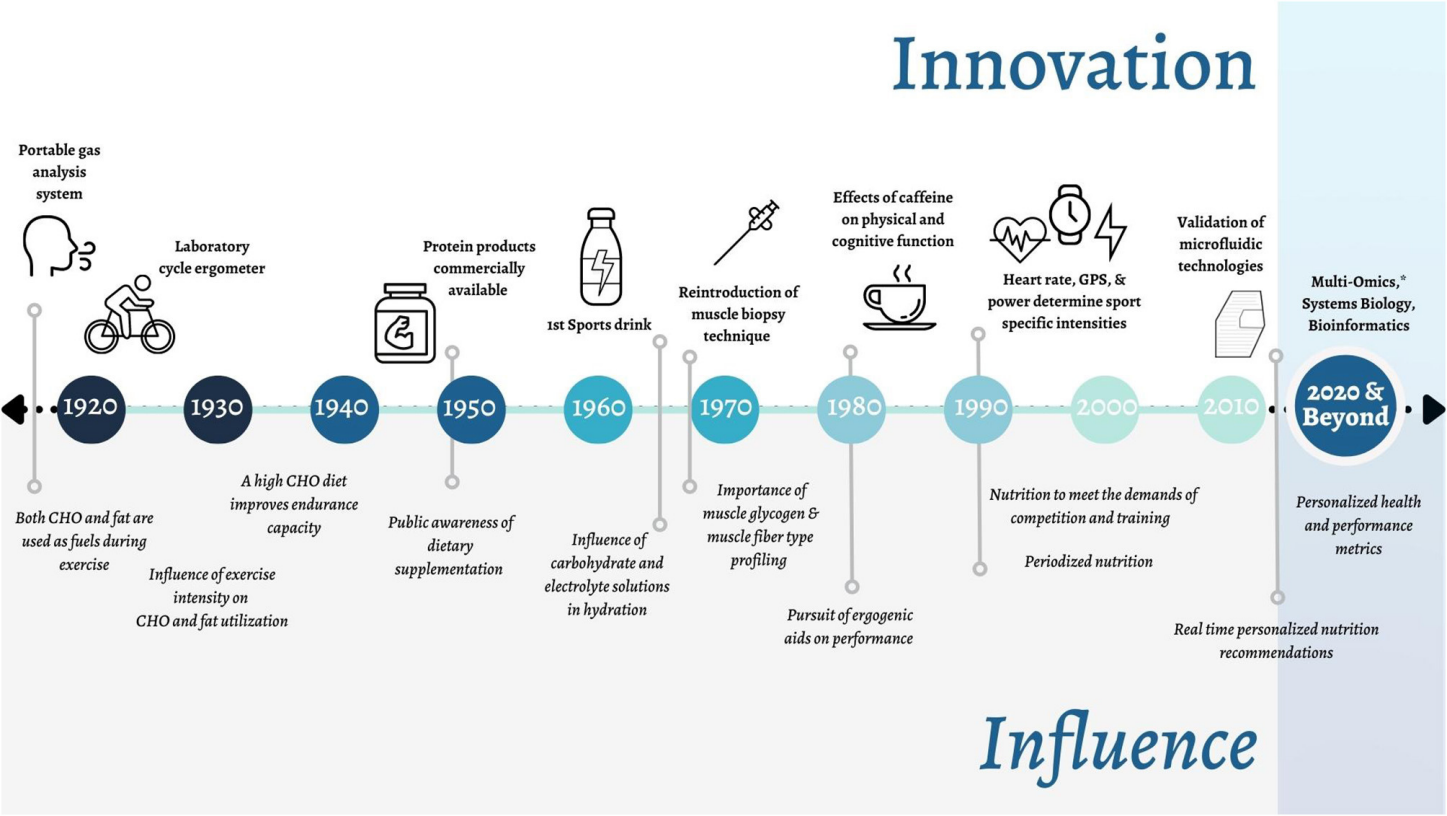
Timeline of key innovations in Sports Nutrition and their respective influence on the field. *Including: genomics, transcriptomics, metabolomics, proteomics, phenomics, and other related omics (e.g., epigenomics).

Innovation has always been at the forefront of sport. Recent examples include drafting in cycling, clap skates in speed skating and more recently, carbon plate shoes in running. Sports nutrition is a relatively young discipline with <100 scientific papers published per year in the early 1990s, to about 3,500 per year today and a myriad of books (Figure 1). Much of this progress was brought about by exercise physiologists who developed new methods and technologies within their laboratories (e.g., treadmills and ergometers) at universities around the world to study trained athletes (e.g., distance runners and cyclists) (Hawley et al., 2015). Next to sports science, these developments promoted the emergence of another new discipline, that of sports nutrition. Some of the major innovations and corresponding knowledge milestones for sports nutrition research, combined with sports science research, are summarized in Figure 2.

In 2003, the International Olympic Committee (IOC) working group on sports nutrition concluded “The amount, composition, and timing of food intake can profoundly affect sports performance. Good nutritional practice will help athletes train hard, recover quickly and adapt more effectively with less risk of illness and injury” (IOC Consensus Statement on Sports Nutrition, 2004). Nearing two decades later and these recommendations remain as pertinent. Despite all this progress, excitement and scientific endeavor, the ability to determine the impact of sports nutrition for different groups of athletes (e.g., different sports, ethnic groups, and sex) is still elusive. For example, there is substantial evidence for carbohydrate (CHO) ingestion before, during and after exercise (Burke et al., 2011; Stellingwerff and Cox, 2014). However, it is difficult to separate the performance benefits of CHO ingestion *per se* vs. all other variables during competition (e.g., environment, competition, technology, equipment, and psychology).

Failure to establish the impact of any sports and exercise science intervention may result in a decline in recognition of the disciplines role in supporting the health and performance of athletes. This applies not only to the elite performers but also the young athlete, the exercising public, and the elderly. Therefore, this mini-review will focus on the opportunities to accelerate knowledge and practice of sports nutrition *via* the integration of technological innovations. We first highlight the present knowledge then propose ways of integrating technical advances and personalized prescription. Particular reference will be given to personalized prescriptions that are transforming and modernizing other life sciences.

## The Present

Although, we primarily think of innovation in sports nutrition as directed at athlete performance outcomes, we also need to be innovative in our methods of synthesizing and dispersing research. The sports nutrition research data generated to date, have been compiled in several consensus documents on general sports (Thomas D. T. et al., 2016), team sports (Collins et al., 2021), and dietary supplements (Maughan et al., 2018). However, a limitation of the current evidence base informing all sport science and sports medicine consensus guidelines is the lack of appropriate intervention studies (e.g., randomized controlled clinical trials) in elite populations (McKay et al., 2022) that are ecologically valid (e.g., in real-life training and competition settings). Furthermore, often in practice, sports nutrition recommendations are retrospective fitted to sports in which the original research was not completed. For example much of the research to inform “stop-and-go” type sports has primarily been completed in soccer (Williams and Rollo, 2015). There is also a primary focus on male subjects in the sports nutrition literature in general; yet most guidelines are “assumed” to be ideal for females as well. Furthermore, in the last 5 years, the focus has shifted from original research to reviews in the area of sports nutrition. Of the published articles, between 17.6 and 20.2% have been reviews (512–570), of which 4.4–6.0 meta-analyses (128–223). Some of this focus on reviews of all types rather than original research is enforced due to restrictions imposed due to Covid-19. However, there is a continuing need for original research in order to advance the discipline.

Consensus meetings and subsequent statements are fundamental in the generation of expert driven guidelines. Over the past 5 years, consensus statements in Sports Nutrition ranged between 0.3 and 0.5% (Williams and Rollo, 2015; Jeukendrup, 2017; Pitsiladis et al., 2017; Sutehall et al., 2018; Burke et al., 2019; Stellingwerff et al., 2019; Baker et al., 2020; Muniz-Pardos et al., 2021) of the published articles. Historically, consensus statements, such as the International Olympic Committee (IOC) consensus on sports supplements (Maughan et al., 2018) are drafted following in-person meetings of leading medical and scientific content experts. However, it is appreciated that such meetings can be costly in terms of budget, time and the carbon footprint of travel. More recently, major consensus statements have implemented remote online approaches, including the entire 2019 World Athletics (formerly IAAF) Nutrition for Athletics Consensus Update (featuring over 40 authors across 17 papers, e.g., 12). A remote consensus approach provides the opportunity to involve a wider contribution on topic guidelines rather than fewer selected opinion leaders. Establishing online working communication platforms as well as documents may also allow consensus documents to be updated with greater frequently or at pace with current literature. In summary, consensus statements informed by original research and meta-analyses, will require a greater reliance on new digital based approaches, while also respecting the need for in person meetings amongst experts.

## Integrating Technical Advances to Field-Based Metrics

Most paradigms in sports nutrition have been established using laboratory experimentation, while neglecting evidence from *in situ* or field experimentation. This results in studies with limited ecological validity. In order to establish the efficacy of nutrition parameters for performance enhancement for all relevant populations, we need to better understand the competition demands of sport (Stellingwerff et al., 2019). Recent advances in wearable technologies and real-time monitoring have accelerated the shift in research from the laboratory to the field in order to enhance ecological validity. This trend poses a real opportunity for all sport science disciplines, including sports nutrition, to embrace these technological developments. One such recent example was the implementation of live performance feedback of athletes (during 10,000 m, marathon, and race-walk events) competing in the heat at Tokyo 2020 (Muniz-Pardos et al., 2021). Briefly, the aim of implementing this wireless technology during Tokyo Olympic Games was to help characterize the physiological and thermal strain experienced by athletes, as well as determine future management of athletes during a medical emergency as a result of a more timely and accurate diagnosis. The real-time monitoring comprised a smartwatch application, designed to collect, process and transmit a wide range of physiological, biomechanical, bioenergetics, and environmental data. This project was a success in terms of technological innovation but also general acceptance by athletes and sport's governing bodies. Such projects provide the opportunity for other new and valid sensors to assess performance- and health-related parameters particularly relevant to sports nutrition. One example is microfluidic technologies integrated into wearable patches to provide athletes instant feedback on sweat rate and sweat composition (Baker et al., 2020). Wider adoption of such technologies will create more symbiotic relationships between sport, health and technology by harnessing the unique demands of elite sport (e.g., the need for unobtrusive devices that provide real-time feedback).

Given their symbiotic relationship, the evolution of sports nutrition, and sports science requires more holistic approaches with input from all major disciplines (e.g., coaching science, environmental physiology, and sports biomechanics), stakeholders, sponsors, and interested industry (Pitsiladis et al., 2017). In recent years, physiology, nutrition, and technical advances have become increasingly integrated as part of new sport performance innovation strategies. A pertinent example is the Sub2 marathon project which was a novel proof-of-concept idea motivated by the need to focus on a holistic approach whilst promoting clean sport (i.e., high performance marathon running without doping) (Pitsiladis et al., 2017). This was the first dedicated international research initiative launched in 2014 made up of multidisciplinary scientists from academia, elite athletes and strategic industry partners across many sport science and medicine domains. An exciting Sub2 innovation with particular sports nutrition focus was the carbohydrate “hydrogel” development. This innovative concept in sports drinks was tested in elite athletes training in Ethiopia and Kenya. The novel aspect of the gel was that it allowed runners to ingest and tolerate CHO concentrations much higher than would normally be possible to ingest while running (e.g., 30% CHO) (Sutehall et al., 2018). This was important because a common challenge for runners is to meet CHO ingestion guidelines without experiencing gastrointestinal complaints (Jeukendrup, 2017). This sports drink was subsequently trialed and tested in the field with positive response by elite runners during marathons and cyclists in the tour de France (Sutehall et al., 2020). One laboratory-based study has confirmed improved running performance, greater carbohydrate oxidation and lower GI symptoms following hydrogel ingestion compared with a standard CHO solution (Rowe et al., 2022). However, other laboratory-based studies have not reported any of these advantages following hydrogel ingestion compared to the ingestion of carbohydrate-electrolyte sport beverages (Baur et al., 2019; King et al., 2020; McCubbin et al., 2020). Nevertheless, it is a great example of sports nutrition innovation specific to the needs of the sport in the field. The hydrogel innovation was adopted by both the breaking^2^,^3^. and INEOS 159^4^. projects to break the 2-h marathon barrier, a reflection of the perceived value of this putative innovation.

Combining emerging technologies are ideal to better our understanding of performance and to objectively test the impact of nutritional strategies in laboratory or real performance environments (Table 1). Such innovations will also allow other sports, beyond the mainly studied endurance sports cycling and running, to be evaluated in terms of sports nutrition impact. The utilization of these technologies, and co-ordinated research, may allow for the rapid generation of large data sets across many other types of sport that have yet to be included in sports nutrition research. As such, this approach will (i) speed our knowledge of sports that are difficult to study, (ii) gain data from regional populations under-represented in the literature, and (iii) inform the advice of how specific nutrition guidelines maybe transferred to the field. Accordingly, Table 1 highlights examples of existing and emerging technologies and methodologies that are “field-based” and relatively non-invasive that may continue to drive and refine sports nutrition research, interventions and recommendations.

**Table 1:** Examples of existing or potential “in field” non-invasive technologies or methodologies that may drive current or future nutrition studies, interventions, and/or recommendations.

**Theme**	**Innovation**	**Potential Nutrition Impact/Recommendations**	**References**
Methodologies	Microfluidic technologies integrated into wearable patches	Instant feedback on sweat rate and sweat composition impaciting on hydration intake and fluid composition	Baker et al., 2020
	Dual-energy X-ray absorptiometry (DXA)	Reference standard to measure BMD	Nieves et al., 2010
	Double labeled water (bolus of 2H218O water, and urinary collection)	Gold standard methodology for measuring free-living total daily energy expenditures, which can impact on projected energetic nutrition requirements	Speakman and Hambly, 2016
	[15N]glycine (bolus of tracer and urinary collection)	Whole-body protein turnover (synthesis, breakdown, and net protein balance) can be calculated by measurement of the excretion rates of 15 N in urinary urea and ammonia	Duggleby and Waterlow, 2005
	Urinary ketone assessment	Ability to better assess CHO availability and/or ketogenic adherence to adjust CHO intake as required	Goffinet et al., 2017
	Urinary specific gravity assessment	It is an estimate of urine osmolality and hydration status	Surapongchai et al., 2021
	Continuous glucose monitoring assessment	Determine the dynamics of blood glucose concentration	Thomas F. et al., 2016
	Biomarkers	Tracking health, performance, and recovery in athletes	Lee et al., 2017
	Isotopic techniques	The study of metabolic flux using stable isotope labeled substrates	Reisz and D'Alessandro, 2017
	DNA and RNA sequencing of DNA and RNA	The process of determining the nucleic acid sequence to identify genes and gene expression (e.g., responders vs. non-responders)	Shendure et al., 2019
Food science	Glucose:Fructose sports drink formulations	Increase the amount of transportable and oxidizable CHO to increase endurance performance and decrease GI issues	Jeukendrup, 2010
	CHO hydrogel sports drink formulations	Increase the amount of gastric emptying resulting in increased amount of transportable and oxidizable CHO to increase endurance performance and decrease GI issues	Sutehall et al., 2018, 2020; Rowe et al., 2022
	Slow-release beta-alanine (containing cellulose type of excipient)	Decrease the amount of urinary losses of beta-alanine as well as decrease the paraesthesia side-effects of beta-alanine as a nutrition ergogenic aid	Décombaz et al., 2012
Sport science/equipment integration	Ergometer power meters	Ability to more accurately estimate exercise energy expenditure to better project energetic nutrition requirements	Haakonssen et al., 2013
	Basic integrated activity monitors (HR, GPS, and accelerometry)	Ability to more accurately estimate exercise energy expenditure and exercise intensity to better project energetic nutrition requirements	O'Driscoll et al., 2020
	Advanced integrated activity monitors (HR, GPS, accelerometry, core, and skin temperature)	Ability to more accurately estimate in real-time exercise energy expenditure, exercise intensity and core body temperature to better project energetic nutrition, hydration and cooling requirements coupled to pacing decisions	Muniz-Pardos et al., 2021

*Some identified technologies/methodologies continue to have construct validity challenges and require further research validation*.

*BMD, bone mineral density; CHO, carbohydrate; GI, gastro-intestinal; GPS, global positioning system; HR, heart-rate*.

The wearable/technological revolution promises in the near future to improve the ability to monitor a whole range of physiological parameters in the field. For example, apps, devices, and entire ecosystems are being developed and destined to improve the quality of dietary intake methods and therefore the accurately of athletes' daily energy intake (EI) (Ferrara et al., 2019). These technological developments may enable the energy availability (EA) of individual athletes (i.e., calculated as EI–EEE/fat free mass) to be more accurately monitored. Correspondingly, a more comprehensively study of the athlete *in situ* would be possible. Thus, this approach represents an unprecedented opportunity to mitigate many unresolved issues in the field of sports nutrition such as relative energy deficiency in sport (RED-S) (Mountjoy et al., 2018). Importantly, the recent explosion of wearable technology/apps/devices with often unsubstantiated claims require quality assurance standards for wearable devices. Such concerns have prompted the International Federation of Sports Medicine (FIMS) to create a global standard for wearable devices in sport and fitness (Ash et al., 2020, 2021). Organizations involved in sports nutrition also have the opportunity to engage in quality assurance processes to safeguard the credibility of the innovations in sports nutrition.

## Personalized Prescriptions

There is no such thing as an “average” athlete. However, a key question is if there is an added value of personalized nutrition vs. general guidelines? Importantly, technology innovations will allow the individual response to a sports nutrition intervention to be determined. For instance, to find the individual recommendation of carbohydrate and fluid during exercise, we need knowledge about the energy demands of the sport, sweat losses, gastrointestinal limits, personal taste preferences and every element of the event. This needs to include research on different sport categories and target groups. This also presents the opportunity to follow athletes over a longer period of time, without associated human labor or time costs. For instance, to establish the extent to which an individual responds to different nutritional interventions, we need to conduct repeated testing in the same individual on several occasions. And the more complex the sport and its environment, the more test repetitions may be needed to establish the magnitude of impact of an intervention. It is also imperative that we determine athlete compliance with prescribed nutritional interventions. Such data will allow the evaluation of education and behavior change strategies, which may also provide opportunity for personalization.

The research on personalized sports nutrition will undoubtedly be the focus in the near future due to the technological advances in genomics technologies such as genetic sequencing. For instance, is has been suggested that the impact of DNA sequencing will become on a par with that of the microscope (Shendure et al., 2019). Sports nutrition and sports science are encouraged to use these powerful technologies and to keep up with rapid developments to increase the chances of finding the best solutions possible. Such technologies are routinely used in biomedical research and precision medicine applications, such as for cancer, stroke and Alzheimer's disease, thus, vital lessons can be learned and transferred to sports nutrition. However, it is essential that these technological developments are not “oversold” and that their application in the field is founded on evidence-based research and not driven by commercial interests. At present, the use of genetic testing in both sports nutrition and sports science is at a very early stage. The consensus in the scientific literature being that genetic testing in sport science has very low clinical utility and should not be sold (Guasch-Ferré et al., 2018; Tanisawa et al., 2020). This is in contrast to the ever-increasing number of companies selling genetic testing, supported by unfounded claims (Webborn et al., 2015; Vlahovich et al., 2017; Tanisawa et al., 2020). The market value of genetic testing; USD 10.80 Billion in 2020, is forecast to reach USD 23.14 Billion by 2027^5^.

A more precision-based sports nutrition will also need to consider the other components of the “omics” cascade in addition to genomics (e.g., transcriptomics, metabolomics, proteomics, and single cell sequencing). Furthermore, such approaches may utilize powerful bioinformatics methods, such as machine learning and artificial intelligence to integrate the different layers of biological data required for understanding the functional consequences with the real time assessment of the “phenome” using 5G and 6G, sensors, devices and applications (Mancin et al., 2021). The identification of relevant non-invasive biomarkers are attractive to athletes and practitioners, due to the speed and increased frequency of collection vs. traditional blood draws or questionnaires. However, these technologies should be adopted in accordance with ethical principles and within national/international regulatory frameworks, which require further development.

## New Approaches to Fulfill Knowledge Gaps

Given recent technological breakthroughs, there are exciting opportunities for sports nutrition research to take gigantic leaps in the near future. Until now, most sports nutrition and sports physiology studies are performed in controlled laboratory environments and often study the effect of single nutrients. There is opportunity for sports nutrition research to embrace real world settings using real solutions and a more holistic approaches, such as performance benefits of whole foods, whole-body effects of low EA and “targeted nutritional periodization.” One example is a study using tracer technologies to compare the effect of whole eggs vs. egg whites on post exercise muscle protein synthesis (van Vliet et al., 2017). New study designs should focus on real life settings that are strictly monitored with use of new technological advances, apps, and systems. As such, with a clear overview of nutritional demands of the sport and individual factors of impact, the extent of real-life effects of sports nutrition elements can be established.

Beyond the physiological impact of nutrients, there is also opportunity for sports nutrition research to study of cognitive and mental performance (Habay et al., 2021). This shift will require sports nutrition researchers and nutritionists to adopt and further develop technological methods to allow the psychobiological determinants of performance to better defined. New research paradigms and technologies could revolutionize sports nutrition research from small landmark studies of the 1960s with mainly the authors as subjects taking muscle biopsies on themselves (Bergström and Hultman, 1966), to the use of big data and collaboration between large groups of researchers. Examples of the latter are studies identifying genes implicated in hand grip strength involving over 195,000 subjects (Willems et al., 2017) or investigating the effects of age, body composition, and sex on total expenditure by the doubly labeled water method in 6,421 participants from 29 countries (Pontzer et al., 2021). The field of sports nutrition has the opportunity to adopt such collaborative practices combined with the application of the new and established technologies (see Table 1). It is reasonable to suggest that this approach will inevitably become the mainstay of personalized medicine, where treating the individual will be the norm rather than the average. If sports nutrition can embrace these challenges, it will thrive as an essential discipline and its relevance recognized in other fields (Oikawa et al., 2021).

## Limitations/Perspectives

While innovations are necessary and appealing, there needs to be a considered approach to implementation. Soon almost any parameter will be able to be measured or inferred, yet the use of such data especially during live performances remains to be explored. There also seems to be a trend toward 24/7 observations (e.g., Apple watch, Oura ring, WHOOP, and Biostrap). Caution is encouraged when moving from too little or no assessment to over monitored and scheduled, as a result of too much feedback and reliance on devices. For instance, the athlete should be focussing on racing/competition, not on heart rate or temperature or non-validated feedback directly from a device. Tracking may also be potentially stressful (Andersen et al., 2020), albeit this remains to be determined in athlete populations. When evolving sports nutrition research with new technological advances, it is important to continuously question the application to practice as well as the reliability and reliance of devices.

The integration of new technologies in elite populations will also require closer collaborations between research and practitioners, and then directly to the athlete and coach (Bartlett and Drust, 2021). However, multidisciplinary sport science and medicine teams do not come without challenges and clear communication, roles and responsibilities are essential (Dijkstra et al., 2014) with the athlete and coach at the centre of accountability.

Finally, impactful implementation of these innovations and technological developments especially in elite athletic populations is going to require the continued and better integration of behavioral change psychology in sports nutrition. A recent systematic review highlighted some of the most effective behavioral strategies used in sports nutrition (Bentley et al., 2020).

## Conclusions

Innovation is at the core of sports nutrition research and has pushed the field forward even before sports nutrition was recognized as a separate discipline. We are at a critical juncture in the evolution of this discipline primed to utilize new technologies to support the success of specific sports and individual athletes. Sharing data in new and more efficient ways, integrating field based physiological measures, and personalized prescriptions are key opportunities to advance sports nutrition. However, technological advances should not be used in haste and must first be evaluated to determine their functionality and value to the athletes health and performance. In summary, nutrition is but one of many complex and integrated sport performance determinants, and the impact of any new intervention should be assessed along a risk-reward continuum.

## Author Contributions

All authors listed have made a substantial, direct, and intellectual contribution to the work and approved it for publication.

## Author Disclaimer

The views expressed in this article are those of the authors and do not necessarily reflect the position or policy of PepsiCo, Inc.

## Conflict of Interest

IR and MK are employees of the Gatorade Sports Science Institute, a division of PepsiCo, Inc. KJ, TS, and YP, received speaking honoraria, for the GSSI ECSS 2021 pre-conference symposium which inspired this article. YP is the founding member of the Sub2 project (www.sub2hrs.com). The Sub2 project is affiliated to a non-trading company (Athlome Limited, UK) that is minor (<1.1%) shareholder of Maurten AB, Gothenburg, Sweden.

## Publisher's Note

All claims expressed in this article are solely those of the authors and do not necessarily represent those of their affiliated organizations, or those of the publisher, the editors and the reviewers. Any product that may be evaluated in this article, or claim that may be made by its manufacturer, is not guaranteed or endorsed by the publisher.

## Footnotes

^1^https://pubmed.ncbi.nlm.nih.gov/?term=sports+nutrition&filter=years.1946-2021&timeline=expanded&sort=date&sort_order=asc.

^2^www.runnersworld.com/uk/news/a29100149/breaking2.

^3^www.nike.com/gb/running/breaking2.

^4^http://www.ineos159challenge.com/.

^5^https://brandessenceresearch.com/requestSample/PostId/1362.
